# MEK5-ERK5 pathway associates with poor survival of breast cancer patients after systemic treatments

**DOI:** 10.18632/oncoscience.135

**Published:** 2015-02-20

**Authors:** Mariska Miranda, Esdy Rozali, Kum Kum Khanna, Fares Al-Ejeh

**Affiliations:** ^1^ QIMR Berghofer Medical Research Institute, Herston QLD, Australia

**Keywords:** ERK5/MAPK7, MEK5/MAP2K5, triple negative breast cancer, TNBC, chemo-resistance

## Abstract

The MEK5-ERK5 pathway is a mammalian mitogen-activated protein (MAP) kinase cascade that is not well studied compared to other MAP kinase cascades. Two independent studies by Al-Ejeh *et al.* and Ortiz-Ruiz *et al.* published in Oncotarget last year concluded that ERK5 is an attractive target in triple negative breast cancer. In this perspective, we briefly describe the findings of these studies and propose the use of pharmacological inhibition of ERK5 in combination with chemotherapy against triple negative breast cancer because MEK5-ERK5 overexpression associates with poor survival of patients treated with chemotherapy.

ERK5 (MAPK7) consists of the N-terminal kinase domain that is highly homologous to the more studied MAP kinase ERK1/2. However, ERK5 - also called the big mitogen activated protein kinase 1 (BMK1) - contains a unique large C-terminal domain which does not exist in other MAP kinases [1, 2]. Two articles published in Oncotarget in 2014 characterized ERK5 as a therapeutic target against triple negative breast cancer [3, 4]. Using the Kinex^TM^ antibody arrays to profile primary breast tumors, we found that a subset of triple negative breast cancer (TNBC) overexpress ERK5 and its upstream activator MEK5. TNBC cell lines were sensitive to the specific ERK5 inhibitor (XMD8-92) and the MEK5/ERK5 inhibitor (BIX02188). We found that the ERK5 inhibition alone and more so with anthracycline/taxane-based chemotherapy had an anti-tumour response against TNBC tumors *in vivo*. This anti-tumor response was associated with increased DNA damage and apoptotic cell death [3]. Ortiz-Ruiz *et al.* [4] reported the overexpression of ERK5 in TNBC and that its inhibition with the CDK/ERK5 inhibitor TG02 [5] induced apoptotic cell death *in vitro* and with an anti-tumor effect *in vivo*. The authors also reported that ERK5 inhibition potentiated chemotherapy *in vitro*. In both studies, members of the Bcl-2 family were modulated by ERK5 inhibition explaining the anti-tumor effect of ERK5 inhibitors [3, 4].

Ortiz-Ruiz *et al.* [4] interrogated published gene expression data from breast cancer using the Kaplan-Meier Online Tool (KM Plotter) [6] and found that ERK5 mRNA overexpression associated with poor relapse-free survival in node-positive basal-like and HER2-enriched breast cancers. While we could replicate their finding, we want to take this opportunity to clarify that the prognostication by ERK5 mRNA is in fact related to chemotherapy rather than node-positivity. We used all the probes which detect mRNA transcripts of ERK5 and found that the stratification of relapse-free survival (RFS) and distant-metastasis-free survival (DMFS) by ERK5 mRNA is relevant to both node-negative (N0) and node-positive (N1) ER-negative tumors only after chemotherapy (Table 1A). We also analyzed the association of ERK5 mRNA with patient outcome in basal-like breast cancer. We found that ERK5 mRNA overexpression associates with poor RFS in patients who received chemotherapy, but not in patients who did not receive chemotherapy (Table 1B). Moreover, we found that considering the overexpression of MEK5 mRNA in addition to ERK5, as an indicator of the MEK5-ERK5 signaling axis, associated with poorer RFS and DMFS in basal-like breast cancer who received chemotherapy (Table 1B and Figure 1A). The combined MEK5-ERK5 mRNA expression also associated with DMFS of HER2-enriched patients who received systemic treatment but not those who were systemically untreated (Figure 1B). Our *in silico* analyses suggest that ER-negative and the basal-like and HER2-enriched intrinsic subtypes of breast cancer with low expression of MEK5-ERK5 do benefit from systemic treatments including chemotherapy, whereas patients with high expression of MEK5-ERK5 do not. It is noteworthy that ERK5 protein expression has been previously associated with poorer survival in breast cancer [7].

**Table 1:** ERK5 mRNA associates with outcome after chemotherapy

A		No chemo				Chemo			
		N0		N1		N0		N1	
		*HR*	*p*	*HR*	*p*	*HR*	*p*	*HR*	*p*
**RFS**	35617_at	*0.85*		*0.16*		9.593	0.0068	3.571	0.0043
	207292_s_at	*0.64*		*0.78*		11.19	0.0035	2.537	0.0346
**DMFS**	35617_at	*0.54*		*0.27*		9.900	0.0236	6.806	0.0034
	207292_s_at	*0.70*		*0.76*		9.251	0.0272	*0.25*	

B		RFS				DMFS			
		No chemo		Chemo		No chemo		Chemo	
		*HR*	*p*	*HR*	*p*	*HR*	*p*	*HR*	*p*
**ERK5**	35617_at	*0.93*		4.0460	0.0027	*0.46*		*0.06*	
	207292_s_at	*0.44*		3.3230	0.0012	*0.97*		*0.06*	
**MEK5**	204756_at	*0.65*		*0.14*		*0.71*		*0.48*	
	210482_x_at	*0.52*		*0.06*		*0.63*		*0.14*	
	211370_s_at	*0.59*		*0.07*		*0.51*		*0.29*	
**MEK5-ERK5**	Combined	*0.51*		3.4970	0.0047	*0.62*		9.9860	0.0485

The KM Plotter Online Tool was used to investigate relapse-free survival (RFS) and distant metastasis-free survival (DMFS) in ER-negative (A) and basal-like (B) breast cancers. In A, patients were also separated according to nodal status (node-negative N0; node-positive N1). Hazard ratio (HR) and p-value (p) from log-rank (Mantel-Cox) test for each analysis were obtained using GraphPad® Prism by comparing patients with the highest expression (top 33%; upper tertile) to those with the lowest expression (bottom 33%; lower tertile). For the MEK5-ERK5 analysis, the average of all the specified probes, which detect mRNA transcripts of MEK5 and ERK5, were used.

**Figure 1:**
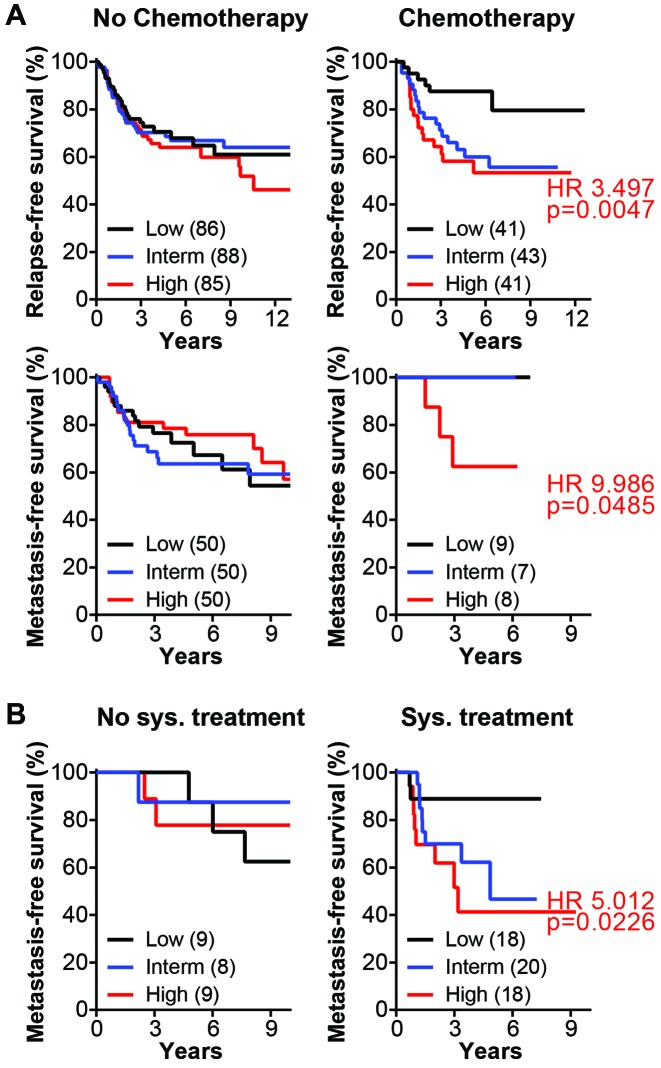
MEK5-ERK5 mRNA expression associates with poor survival after systemic treatments (A) Relapse-free survival (top row) and distant metastasis-free survival (middle row) of basal-like breast cancer were analyzed in subsets of patients who did not receive or received chemotherapy (No chemotherapy vs. Chemotherapy). (B) Distant metastasis-free survival of HER2-enriched breast cancer in subsets of patients who did not receive systemic treatments or received systemic treatments (No sys. treatment vs. Sys. treatment). The KM Plotter Online Tool was used to carry out the analyses. Patients were stratified according to the average expression level of MEK5 and ERK5. The number of patients in the lower tertile (low; bottom 33%), the upper tertile (high, top 33%) and the middle tertile (intermediate; interm) groups are indicated in parentheses. HR and log-rank p-value (p) for each analysis were obtained using GraphPad^®^ Prism by comparing the survival of patients in the upper tertile (High) to those in the lower tertile (Low).

In conclusion, we [3] and Ortiz-Ruiz *et al.* [4] found that ERK5 inhibition potentiates chemotherapy *in vitro* and *in vivo*. These findings along with the lack of benefit from chemotherapy in patients with MEK5-ERK5 overexpression support the rationale to inhibit MEK5-ERK5 signaling pathway in combination with neoadjuvant and/or adjuvant chemotherapy in ER-negative, TNBC and basal-like breast cancer to improve survival rates.
